# Progress and Current Status in Hajdu-Cheney Syndrome with Focus on Novel Genetic Research

**DOI:** 10.3390/ijms231911374

**Published:** 2022-09-27

**Authors:** Natsuko Aida, Tatsukuni Ohno, Toshifumi Azuma

**Affiliations:** 1 Department of Biochemistry, Tokyo Dental College, 2-9-18 Kandamisaki-cho Chiyoda-ku, Tokyo 101-0061, Japan; 2 Oral Health Science Center, Tokyo Dental College, 2-9-18 Kanadamisaki-cho Chiyoda-ku, Tokyo 101-0061, Japan

**Keywords:** Hajdu-Cheney syndrome, *NOTCH2*, osteoporosis, bone disease, genes in bone tissues

## Abstract

Hajdu-Cheney syndrome (HCS) is a rare autosomal dominant manifestation of a congenital genetic disorder caused by a mutation in the *NOTCH2* gene. NOTCH signaling has variations from NOTCH 1 to 4 and maintains homeostasis by determining and regulating the proliferation and differentiation of various cells. In HCS, the over-accumulated *NOTCH2* causes abnormal bone resorption due to its continuous excessive signaling. HCS is characterized by progressive bone destruction, has complex wide-range clinical manifestations, and significantly impacts the patient’s quality of life. However, no effective treatment has been established for HCS to date. There are genetic variants of *NOTCH2* that have been reported in the ClinVar database of the U.S. National Institutes of Health. In total, 26 mutant variants were detected based on the American College of Medical Genetics and Genomics (ACMC). To date, there has been no comprehensive compilation of HCS mutations. In this review, we provide the most comprehensive list possible of HCS variants, nucleotide changes, amino acid definitions, and molecular consequences reported to date, following the ACMC guidelines.

## 1. Background and Clinical Manifestations

Hajdu-Cheney syndrome (HCS) is a rare autosomal dominant manifestation of a congenital genetic disorder. It was first described by Hajdu and Kauntze in 1948 [1]. In 1965, Cheney reported a familial form of the disease [2,3]. HCS is registered in the OMIM (Online Mendelian Inheritance in Man) project database with reference #102500 and in ORPHANET under the reference ORPHA955. The prevalence of HCS is less than 1 in 1,000,000 making it an extremely rare genetic syndrome. The gender or racial differences in HCS prevalence remain unclear. HCS is characterized by resorption of the distal phalanges of the feet and in the fingers, often with inflammatory epiphyseal lysis, which causes pain and swelling. Progressive bone destruction, especially severe osteoporosis, is also found, including spinal abnormalities such as compression fractures and deformities, as well as craniofacial deformities. Moreover, cardiac diseases such as cardiovascular abnormalities and valvular insufficiency as well as dental issues, including abnormal redness of the gingiva, caries, severe periodontal disease, premature tooth loss, cleft palates, and abnormal tooth eruption, are also observed in HCS [1,4]. Neurological disorders and polycystic kidney disease may also occur [5]. In most patients with HCS, mental development is reported to progress at a normal rate [2]. Overall, Hajdu-Cheney Syndrome has complex clinical manifestations. The significant features in patients with HCS are listed below (Table 1).

Thus, HCS is a severe genetic disorder with wide-ranging clinical manifestations and a significant impact on the patient’s quality of life. Furthermore, patients diagnosed with HCS have a variable clinical presentation that varies from early infancy to late adulthood, worsening over time because of age-dependent progression [6,7]. The complex clinical symptoms, which vary from patient to patient, hinder the diagnosis of HCS based on patient clinical features alone. Further, as this disease is extremely rare, no clinical trials with statistical analysis have been conducted, and the accumulation of clinical reports is extremely insufficient. Therefore, no effective treatment has been established for HCS to date. This review summarizes the mutations associated with HCS, and its clinical management along with recent work in animal models to provide potential insights into the treatment of this rare disease.

## 2. NOTCH Signaling

In 2011, a mutation in the *NOTCH2* gene on chromosome 1 (locus 1p13-p11) was identified as a causative gene in patients with HCS based on whole exome analysis [8,9].

NOTCH signaling is an evolutionarily conserved pathway in multicellular organisms, which maintains homeostasis in living tissues by determining and regulating the proliferation and differentiation of various cells during development. Loss of function in NOTCH is known to cause Adams-Oliver syndrome, Alagille syndrome, spondylocostal dysostosis, and congenital cardiac disease. In contrast, gain of function in *NOTCH* causes HCS, serpentine fibula polycystic kidney syndrome, infantile myofibromatosis, and lateral meningocele syndrome [8,9,10,11,12,13].

**Figure 1:**
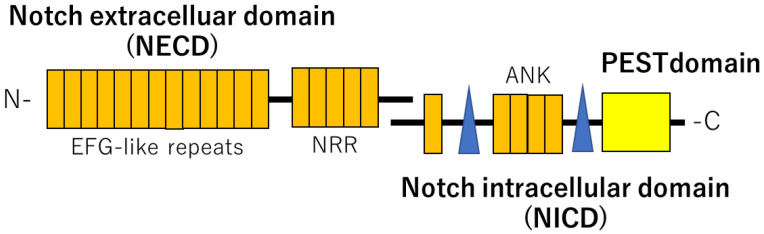
An overview of NOTCH signaling, and its involvement in Hajdu-Cheney Syndrome.

The NOTCH pathway is described as a cell-contact signaling pathway that requires physical contact between adjacent cells in a short-range. NOTCH proteins are heterodimeric, single transmembrane proteins functioning as receptors for DSL (Delta, Serrate, Lag2) family ligands. They are divided into three major parts: the extracellular domain (NECD), the transmembrane domain, and the intracellular domain (NICD). The extracellular domain, which is the receptor part, is transported to the cell surface and exits the cell by exocytosis, allowing it to bind ligands. There are four variants of NOTCH (NOTCH1-4) and their ligands include five known proteins (JAG1, JAG2, DLL1, DLL3, DLL4) on the adjacent cell surface [14,15]. NOTCH protein is activated by binding to one of these ligands. The intracellular domain of NOTCH (NICD) is then detached and transported into the nucleus, where it forms a complex with a DNA-binding protein called CSL to induce the expression of downstream target genes (Figure 1).

The NOTCH receptor consists of the NOTCH extracellular domain (NECD) and NOTCH intracellular domain (NICD). Mutations of *NOTCH2* cause the deletion of the PEST domain in the NICD, which results in *NOTCH2* overexpression, leading to the Hajdu-Cheney syndrome.

## 3. Gene Mutation in *NOTCH2*

Isidor B. et al. reported that a genetic mutation in *NOTCH2* causes HCS [9]. Further reports indicated that a frameshift in exon 34, which is located upstream of the PEST (proline–glutamic acid–serine–threonine-rich) domain in the final exon on the C-terminal side of *NOTCH2*, results in deletion of the sequence the PEST domain. The PEST domain regulates NICD stability by its ubiquitination. The ubiquitination of PEST promotes proteolysis of the NICD domain. Thus, deletion of the PEST domain prolongs the survival of NICD and results in excessive NOTCH signaling. The *NOTCH2* signaling pathway plays an important role in osteoclast differentiation and in the promotion of osteoclastogenesis [16]. Deviating from proteasome-dependent proteolysis via ubiquitination by the SCF^FBW7^ ubiquitin ligase complex, non-degraded stable *NOTCH2* mutants accumulate in excess. The over-accumulated *NOTCH2* involved in osteoclast differentiation continues to signal excessively, which enhances osteoclast differentiation and causes abnormal bone resorption.

Another mechanism for excessive bone resorption caused by the overactivation of NOTCH signaling is through increased tumor necrosis factor-alpha (TNFα), which may promote osteoclast differentiation [17].

In HCS, the proto-oncogene c-Fos (c-FOS) and nuclear factor of activated T cells 1 (NFATc1) show increased expression, which promotes osteoclast differentiation. HCS is a unique disorder that causes inflammation and bone destruction simultaneously, along with systemic osteolysis and osteoporosis. Therefore, it is necessary to elucidate why osteolysis is accompanied by inflammation and the pathogenesis of HCS.

## 4. Variants of the *NOTCH2* Gene

High-throughput next-generation sequencing has led to remarkable advances in genetic analysis over the past decade. Simultaneously, the analysis of genetic disorders has become more complex, and base sequence differences (variants) between various individuals have been demonstrated to be more abundant than previously expected [18]. In 2015, the American College of Medical Genetics and Genomics (ACMC), in collaboration with the Association of Molecular Pathology (AMP) and the College of American Pathologists (CAP), developed standards and guidelines for sequence interpretation of variants in this genetic analysis [19]. These guidelines established specific criteria to define the variants identified in genes responsible for Mendelian diseases, including “Pathogenic”, “Likely pathogenic”, “Uncertain significance”, “Likely benign”, and “Benign”. Further, by evaluating multiple criteria in relation to pathogenicity, and by synthesizing each evaluation to ultimately determine whether the disease is pathogenic, these evaluation methods facilitate objective and accurate judgment.

We examined the genetic variants of *NOTCH2* that have been reported to date and aggregated in the ClinVar database of the U.S. National Institutes of Health. In total, 377 mutant variants were detected; however, our analysis was conducted with the “Condition” parameter set as “Hajdu-Cheney Syndrome” and “Clinical significance” set as “Pathogenic” based on the ACMC guidelines. To our knowledge, this is the first report to classify Hajdu-Cheney Syndrome variants, nucleotide changes, amino acid definitions, and molecular consequences according to the ACMC guidelines. The variants of the 26 analyzed cases are summarized in Table 2. The variant types included 10 single nucleotide variants, 10 deletions, 2 microsatellites, 2 duplications, and 2 insertions/indels. The molecular consequence indicating a functional effect was 12 cases for nonsense and 13 for frameshift mutations, with one unknown. In summary, the *NOTCH2* mutation that causes Hajdu-Cheney syndrome is a nonsense mutation that replaces a stop codon through a base swap, leading to translation of the transcribed messenger RNA beyond the original gene sequence after the mutation. These results also indicate that the disease onset is attributed to frameshifts in gene translation caused by deletion, duplication, indel, insertion, and microsatellite formation. Taken together, based on the current results, missense mutations by single nucleotide substitutions, which have been thought to be causative, may not cause Hajdu-Cheney syndrome under the pathogenic condition of the guidelines.

## 5. Clinical Treatment

The clinical treatment of patients with HCS is challenging, as no treatment strategies have been established and conventional conservative treatments with drugs and surgical treatment are being used at present. Although HCS is a genetic disease and therefore difficult to treat effectively, the goal of current treatment is to improve the quality of life of patients by reducing complications and alleviating their symptoms [8]. Bisphosphonates (BP) and denosumab are often used to treat osteoporosis, which is characterized by progressive bone resorption [20]. Bisphosphonates are anti-bone resorptive and anti-catabolic agents that have a P–C–P structure. They have an affinity for hydroxyapatite and affect bone turnover [21,22]. Denosumab is a monoclonal IgG2 antibody against RANKL, which inhibits osteoclast generation, and binds to RANKL. RANK, RANKL, and osteoprotegerin, which typically regulate bone remodeling; however, when this pathway is uncontrolled, as in patients with HCS, denosumab has not been applicable [20,23,24]. The off-label use of BP and denosumab is because they are both drugs that may induce drug-related osteonecrosis of the jaw (MRONJ) [25,26].

In the genetic disorder HCS, which causes progressive bone resorption with inflammation, impaired bone growth and remodeling, imbalance between the numbers of osteoblasts and osteoclasts, as well as excessive osteoclast activation are believed to form the underlying mechanism. In patients with HCS, anti-resorptive and anti-angiogenic agents have been used to primarily retard osteoporosis development and distal phalanx bone resorption. BPs have been reported to exert a positive effect on the bone in patients with HCS [27,28], but other studies have failed to confirm this effect. Thus, at present, there is no clear evidence that BPs are beneficial; in fact, it may prevent the initiation of bone formation in humans and may not allow activation of bone remodeling [29]. Denosumab has been shown to increase bone mineral density, but has no effect on osteolysis of the endosteum [20]. However, BPs and denosumab are not indicated for surgical treatment or surgical procedures related to the alveolar bone (extraction, periosteoplasty, etc.), nor for periodontal disease, peri-implantitis, and prosthetic procedures, because of the risk of MRONJ.

Abnormal tooth eruption and premature tooth loss are usual features that are particularly prominent in patients with HCS. Structural hard tissue changes in the cementum and dentin, along with hypoplastic dental roots, result in the premature exfoliation of the teeth [30]. Thus, any interference, stimulation or injury can lead to abnormal healing or osteonecrosis. Further, taking anti-resorptive or anti-angiogenic agents may also be a risk factor [4]. Therefore, the treatment of patients with HCS, in which the inflammatory destruction of bone is more advanced than in patients with osteoporosis, requires further management, prophylactic antibacterial agents, and appropriate diagnostic and radiographic studies in addition to the conventional osteoporosis treatment [4].

## 6. Recent Reports and Genetics of Mouse Models

Osteoporosis is a specific clinical manifestation of HCS, and the NOTCH pathway is assumed to be important in bone tissue homeostasis; however, the role of *NOTCH2* in osteoblast differentiation has been rarely reported [31,32,33]. In recent years, NOTCH-related studies on bone have gradually gained increasing attention [9,34,35]. Engin et al. found that loss of NOTCH signaling is correlated with bone health [36]. However, some papers have reported no relationship between osteoclasts and bone resorption caused by *NOTCH2* hyperaccumulation. In any case, there is currently no consensus on osteoclast activation via the NOTCH pathway [37,38].

In 2016, Canalis et al. created an HCS mouse model by introducing the 6955C>T mutation into exon 34 upstream of the PEST domain of *Notch2* using homologous recombination. This single nucleotide substitution mutation is a nonsense mutation that creates a STOP codon upstream of *Notch2* exon 34, resulting in the expression of a truncated Notch2 protein with loss of PEST domain in the model mouse (*Notch2^Q2319X^*) [39]. *Notch2^Q2319X^* heterozygous mutant mice were smaller and had shorter femurs and reduced trabecular and cortical bone mass in both males and females compared to those in the controls. They showed increased osteoclast numbers and bone resorption in the trabecular bone but no decrease in osteoblast numbers or bone formation. This indicated that *Notch2^Q2319X^* cells did not affect osteoblast differentiation or function [34]. Antibodies generated against the negative regulatory region (NRR) of NOTCH suppressed osteopenia and reduced the osteoclast numbers in mouse trabecular bone. Furthermore, subcutaneous administration of *Notch2* antisense oligonucleotide (ASO) reduced osteoclast numbers and induction of TNF superfamily member 11 (Tnfsf11) mRNA levels in mice, suggesting that systemic administration of *Notch2* ASO can improve the reduction of bone mass in *NOTCH2* model mice [40,41]. Moreover, the study also examined whether the skeleton can be sensitized to the osteolytic effects of tumor necrosis factor α (TNFα). TNFα was injected into the cranium, resulting in an increase in osteoclast numbers and osteoclast surface. However, the inactivation of Hes1 reduced the effect of TNFα on osteoclastogenesis. This indicates that TNFα promotes osteoclastogenesis and inflammatory bone resorption, leading to epiphyseal resorption [17]. Another group has used this mouse model to suggest that HCS mutations increase the susceptibility to osteoarthritis and further reported that *NOTCH2* activation is involved in HCS pathogenesis [42]. These studies demonstrate that HCS-like *NOTCH2* mutant model mice are severely osteopenic with increased bone resorption. They concluded that this is caused by the sensitization of osteoclasts to the receptor activator of nuclear factor-B ligand and the increased expression of tumor necrosis factor superfamily member 11 (Tnfsf11) in osteoblasts. A *NOTCH2* conditioned-by-inversion (*Notch2*^COIN^) model has also been created in which Cre recombination generates a *Notch2^ΔPEST^* allele that expresses a *NOTCH2* mutant lacking the PEST domain. Interestingly, osteopenia was observed when these mice were crossed with mice expressing Cre from the BGLAP or Lyz2 promoter to induce HCS mutation only in osteoblasts and not in osteoclasts. The authors thus noted that the HCS mutation in osteoblasts instead of osteoclasts causes osteopenia [37].

Vollersen et al. introduced another HCS-related mutation (6272delT, Notch2/F2091SsX4) in the mouse *NOTCH2* gene to create an HCS model mouse in 2018 [43]. Although no osteocyte abnormalities were observed in these mice, excessive skeletal remodeling was identified. In addition, trabecular bone loss was observed at all ages; however, acro-osteolysis, a hallmark of HCS, did not occur in these mice. Other phenotypic manifestations, such as renal cysts and craniofacial abnormalities, which vary in clinical manifestation from patient to patient, may not appear as a phenotype. Therefore, these mouse models have several limitations. However, treatment with alendronate prevented the osteopenia phenotype in these mice. Further, the observed high rates of bone formation were also normalized. The increased expression of Tnfs11 and IL6 in osteoblasts of model mice indicates that *NOTCH2* does not regulate osteoblast activity directly but instead exhibits an osteoclast-promoting gene expression pattern, which results in high bone metabolic turnover. Yorgan et al. also observed *NOTCH2* expression in osteoblasts and osteoclasts, and then inactivated *Notch2* in Runx2-Cre and Lyz2-Cre mice. Notch2^fl/fl^/Lyz2-Cre mice showed no significant differences in skeletal growth, bone mass, and remodeling whereas Notch2^fl/fl^/Runx2-Cre mouse showed skeletal abnormalities in long bones. Therefore, *NOTCH2* was considered to suppress trabecular bone formation in the skeleton and to regulate bone remodeling [44]. Furthermore, in a mouse model of HCS, Fukushima et al. reported that *NOTCH2* overexpression in osteoclasts enhances osteoclast function by inhibiting *NOTCH2* degradation [45].

## 7. Conclusions

Although NOTCH signaling is complex and is essential for human survival, studying the effects of its mutations in human genes is very challenging in some respects. Despite its importance, the relationship between NOTCH signaling and bone formation and development is beginning to be addressed only recently. Although it has recently become possible to explore the effects of NOTCH function in humans, for example by creating knockout mice, and to obtain information on genetic diseases, studies in mice have several practical limitations. The generation of disease-specific induced pluripotent stem cells using samples from patients and further research on genetic diseases can provide information that is more relevant to the human body. This information may play a significant role in establishing the pathogenic mechanism of genetic diseases and their treatment. Elucidating the detailed mechanism of this disease and establishing effective treatment methods can thus be expected to improve the quality of life for patients with HCS in the future.

## Figures and Tables

**Table 1:** Clinical features of patients with Hajdu-Cheney Syndrome.

Craniofacial Abnormality	Dental Abnormality	Skeletal Abnormality	Cardiac Diseases	Others
Micrognathism	Highly arched palates	Acroosteolysis	Cardiovascular abnormalities	Polycystic kidneys
Facial dysmorphism	Caries	Fibular deformities, severe osteoporosis	Valvular insufficiency	Neurological disorders
Open sutures, wormian bones	Severe periodontal disease	Severe osteoporosis		
Platybasia and basilar invagination	Premature tooth loss	Fractures		
	Abnormal redness of gingiva	Joint hyperlaxity		
	Abnormal of tooth eruption	Compression fractures and deformities		
		Short stature, developmental delay		

**Table 2:** Classification of Hajdu-Cheney Syndrome variants according to the standards and Guidelines for sequence interpretation of variants of the American College of Medical Genetics and Genomics (ACMC). This classification shows 26 cases of “Pathogenic” variants including nucleotide changes, amino acid definitions, and molecular consequences.

Human Genome Variation Society (HGVS)	Variant Type	Nucleotide Change	Protein (Amino Acid Definition)	Molecular Consequence (Functional Effect)	dbSNP
M_024408.4(NOTCH2):c.1668C>A(p.Cys556Ter)	SNV	c.1668C>A	p.Cys556Ter	nonsense	-
NM_024408.4(NOTCH2):c.2235_2236del (p.Cys745_Asp746delinsTer)	Microsatelite	c.2235_2236del	p.Cys745_Asp746delinsTer	nonsense	-
NM_024408.4(NOTCH2):c.3415del(p.Leu1139fs)	Deletion	c.3415del	p.Leu1139fs	frameshift	-
NM_024408.4(NOTCH2):c.4174C>T(p.Gln1392Ter)	SNV	c.4174C>T	p.Gln1392Ter	nonsense	rs1649449471
NM_024408.4(NOTCH2):c.5123_5132delinsAGA(p.Gln1392Ter)	Indel	c.5123_5132delinsAGA	p.Gln1392Ter	nonsense	rs1649314295
NM_024408.4(NOTCH2):c.5345del(p.Asp1782fs)	Deletion	c.5345del	p.Asp1782fs	frameshift	rs1553193977
NM_024408.4(NOTCH2):c.6272del(p.Phe2091fs)	Deletion	c.6272del	p.Phe2091fs	frameshift	rs1557802353
NM_024408.4(NOTCH2):c.6386del(p.Ser2129fs)	Deletion	c.6386del	p.Ser2129fs	frameshift	-
NM_024408.4(NOTCH2):c.6403_6404del(p.Leu2135fs)	Microsatelite	c.6403_6404del	p.Leu2135fs	frameshift	rs1649067817
NM_024408.4(NOTCH2):c.6424_6427del(p.Ser2142fs)	Deletion	c.6424_6427del	p.Ser2142fs	frameshift	rs1064793515
NM_024408.4(NOTCH2):c.6426_6427insTT(p.Leu2135fs)	Insertion	c.6426_6427insTT	p.Leu2135fs	frameshift	rs1649066485
NM_024408.4(NOTCH2):c.6449_6450del(p.Pro2150fs)	Deletion	c.6449_6450del	p.Pro2150fs	frameshift	rs1553193574
NM_024408.4(NOTCH2):c.6503del(p.Pro2168fs)	Deletion	c.6503del	p.Pro2168fs	frameshift	rs1557802165
NM_024408.4(NOTCH2):c.6622C>T(p.Gln2208Ter)	SNV	c.6622C>T	p.Gln2208Ter	nonsense	rs387906746
NM_024408.4(NOTCH2):c.6832dup(p.Thr2278fs)	Duplication	c.6832dup	p.Thr2278fs	frameshift	-
NM_024408.4(NOTCH2):c.6853C>T(p.Gln2285Ter)	SNV	c.6853C>T	p.Gln2285Ter	nonsense	rs1553193507
NM_024408.4(NOTCH2):c.6877del(p.His2293fs)	Deletion	c.6877del	p.His2293fs	frameshift	rs1649047546
NM_024408.4(NOTCH2):c.6895G>T(p.Glu2299Ter)	SNV	c.6895G>T	p.Glu2299Ter	nonsense	rs387906748
NM_024408.4(NOTCH2):c.6909del(p.Ile2304fs)	Deletion	c.6909del	p.Ile2304fs	frameshift	rs771237928
NM_024408.4(NOTCH2):c.6909dup(p.Ile2304fs)	Duplication	c.6909dup	p.Ile2304fs	frameshift	rs771237928
NM_024408.4(NOTCH2):c.6949C>T(p.Gln2317Ter)	SNV	c.6949C>T	p.Gln2317Ter	nonsense	rs387906747
NM_024408.4(NOTCH2):c.7078C>T(p.Gln2360Ter)	SNV	c.7078C>T	p.Gln2360Ter	nonsense	rs1553193485
NM_024408.4(NOTCH2):c.7090del(p.Gln2364fs)	Deletion	c.7090del	p.Gln2364fs	frameshift	rs1649037695
NM_024408.4(NOTCH2):c.7119T>G(p.Tyr2373Ter)	SNV	c.7119T>G	p.Tyr2373Ter	nonsense	rs1557801639
NM_024408.4(NOTCH2):c.7165C>T(p.Gln2389Ter)	SNV	c.7165C>T	p.Gln2389Ter	nonsense	rs387906749
NOTCH2, 1-BP DEL, 6460T	Deletion	6460T	-	-	-

## Data Availability

Not applicable.
